# Use of Hypotonic Maintenance Intravenous Fluids and Hospital-Acquired Hyponatremia Remain Common in Children Admitted to a General Pediatric Ward

**DOI:** 10.3389/fped.2016.00090

**Published:** 2016-08-25

**Authors:** Shikha Shukla, Srikanta Basu, Michael L. Moritz

**Affiliations:** ^1^Department of Pediatrics, Kalawati Saran Children’s Hospital, Lady Hardinge Medical College, New Delhi, India; ^2^Department of Pediatrics, Children’s Hospital of Pittsburgh of UPMC, University of Pittsburgh School of Medicine, Pittsburgh, PA, USA

**Keywords:** fluid therapy, hyponatremia, hypernatremia, pediatric, hypotonic fluids

## Abstract

**Aim:**

To evaluate maintenance intravenous fluid-prescribing practices and the incidence of hospital-acquired hyponatremia in children admitted to a general pediatric ward.

**Methods:**

This is a prospective observational study conducted over a 2-month period in children ages 2 months to 5 years who were admitted to a general pediatric ward and who were receiving maintenance intravenous fluids. The composition, rate, and duration of intravenous fluids were chosen at the discretion of the treating physician. Serum biochemistries were obtained at baseline and 24 h following admission. Patients who were at high risk for developing hyponatremia or hypernatremia or had underlying chronic diseases or were receiving medications associated with a disorder in sodium and water homeostasis were excluded. Intravenous fluid composition and the incidence of hyponatremia (sodium <135 mEq/L) were assessed.

**Results:**

Fifty-six children were enrolled. All received hypotonic fluids; 87.5% received 0.18% sodium chloride (NaCl) and 14.3% received 0.45% NaCl. Forty percent of patients (17/42) with a serum sodium (SNa) less than 140 mEq/L experienced a fall in SNa with 12.5% of all patients (7/56) developing hospital-acquired or aggravated hyponatremia (126–134 mEq/L) with fall in SNa between 2 and 10 mEq/L.

**Conclusion:**

Administration of hypotonic fluids was a prevalent practice in children admitted to a general pediatric ward and is associated with acute hospital-acquired hyponatremia.

## Introduction

Over the past decade, there has been increasing concern that the routine practice of administrating hypotonic intravenous maintenance fluids to hospitalized children leads to potentially dangerous hyponatremia (1, 2). Hospitalized children have numerous hemodynamic and non-hemodynamic stimuli for arginine vasopressin (AVP) production, which place them at risk for developing hyponatremia (3). Recent meta-analysis evaluating prospective and randomized trials have revealed that hypotonic fluids significantly increase the relative risk for developing hyponatremia in comparison to isotonic fluids (4–6). A recent large retrospective study demonstrated that the incidence of hospital-acquired hyponatremia in children receiving hypotonic fluids is nearly 40% (7). The majority of prospective and retrospective studies evaluating the relationship between hypotonic fluids and hospital-acquired hyponatremia have included high-risk patients, such as patients admitted to the intensive care unit (8), post-surgical patients (9, 10), and patients with gastroenteritis (11), and diverse patient populations, including patients with malignancies and cardiac and renal diseases (7, 12, 13). Studies on fluid-prescribing practices and the incidence of hyponatremia in the general pediatric ward are lacking. To the best of our knowledge, there have been no prospective observational studies evaluating maintenance intravenous fluid-prescribing practices and the incidence of hospital-acquired hyponatremia in children admitted to a general pediatric ward. We suspected that hypotonic fluids are still commonly used and associated with hospital-acquired hyponatremia, despite the significant literature supporting the use of isotonic maintenance fluids.

We conducted a prospective observational study at a tertiary-care hospital in India in order to accurately evaluate maintenance intravenous fluid-prescribing practices and the incidence of hospital-acquired hyponatremia in young children admitted to a general pediatric ward. This study excluded most patients at high risk for developing hospital-acquired hyponatremia.

## Materials and Methods

A prospective observational study was carried out over a 2-month period at Kalawati Saran Children’s Hospital in New Delhi during May and June, 2013. The study was approved by the hospital’s Institutional Ethics Committee and informed consent was obtained for all patients. The study enrolled young children, ages 2 months to 5 years, presenting to the emergency department or to the general pediatric ward, who were anticipated to require at least 24 h of maintenance intravenous fluids (mIVF). All patients were screened, consented, and enrolled by one investigator who was not involved in the management of the patients. The composition, rate, and duration of the mIVF were left to the discretion of the treating physicians who were aware of the study. Patients who were at high risk for developing hyponatremia or hypernatremia, or who had an underlying chronic disease, or who were receiving medications associated with a disorder in sodium and water homeostasis, were excluded. Exclusion criteria included evidence of volume depletion requiring fluid boluses prior to or anytime during the observation periods, patients with gastroenteritis, severe acute malnutrition, postoperative patients, head trauma patients, patients with congestive heart failure, cirrhosis, nephrotic syndrome, acute glomerulonephritis, chronic kidney disease, diabetes mellitus, adrenal insufficiency, or patients who were admitted to the intensive care unit any time during the observation period. Serum biochemistry profile and complete blood count were obtained in all patients at baseline and at 24 h. Patients were excluded if they had moderate hyponatremia or hypernatremia at presentation, serum sodium (SNa) <130 mEq/L or >150 mEq/L. Data regarding admitting diagnosis, intravenous fluid composition, and biochemical parameters were recorded for each patient. For study purposes, hyponatremia was defined as a SNa <135 mEq/L. Any neurologic complications attributable to a fall in SNa were recorded.

Data that were not normally distributed were reported as median, inter-quartile range, and/or range.

## Results

**Figure 1:**
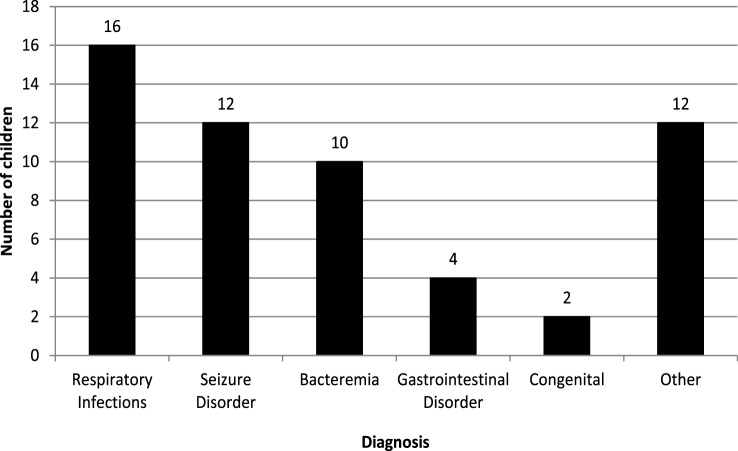
**Primary diagnosis of study population**.

Fifty-six patients successfully completed the study out of 60 patients who screened and consented. Four patients were excluded because electrolytes were not obtained at 24 h. The median age was 1.5 years and 73.2% of the patients were male. The primary reasons for admission are displayed in Figure 1. The most common admitting diagnoses were respiratory infections, seizures, and bacteremia. All patients were administered hypotonic fluids, with 87.5% (49/56) receiving 0.18% saline in 5% dextrose in water and the remaining receiving 0.45% saline in 5% dextrose in water. No child received isotonic mIVF.

The mean SNa at baseline and at 24 h were unchanged, at 138.8 mEq/L. Table 1 analyzes the median change in SNa at 24 h compared with baseline SNa. Fifty percent of the children (28/56) experienced a fall in SNa at 24 h, of which 24 children had a normal or below normal baseline SNa (<145 mEq/L). Seventeen patients with an initial SNa ≤140 mEq/L had a fall in SNa (17/42, 40%) (Bold font in Table 1). Seven patients (12.5%) developed hospital-acquired or hospital-aggravated hyponatremia at 24 h (Table 2), with two patients experiencing large falls in SNa of 9 and 10 mEq/L, while receiving 0.18% saline (Bold font in Table 2). No patients developed symptomatic hyponatremia. The incidence of hyponatremia was no different for those receiving 0.18% saline (6/49, 12%) to those receiving 0.45% saline (1/7, 14%).

**Table 1:** **Change in serum sodium as a function of the presenting serum sodium**.

	Fall in serum Na^+^		No change in Na^+^		Rise in serum Na^+^		Total no. of patients
Serum Na^+^ at 0 h (mEq/L)	No. of patients	Median fall (range) (mEq/L)	No. of patients	Median fall (range) (mEq/L)	No. of patients	Median rise (range) (mEq/L)	
**<135**	**1**	**2**	0	–	3	12 (6,13)	4
**135–140**	**16**	**2.5 (1, 10)**	3	–	19	4 (1, 6)	38
141–145	7	6 (4, 9)	0	–	2	2.5 (2, 3)	9
>145	4	13 (4,14)	1	–	0	–	5
Total no. of patients	28		4		24		56

*Bold font: patients with a serum sodium <140 mEq/L who experienced a fall in serum sodium*.

**Table 2:** **Clinical and biochemical characteristics of patients with hospital-acquired or -aggravated hyponatremia**.

	Age (years)	Diagnosis	Fluid received (%NaCl)	0 h serum Na^+^ (mEq/L)	Blood urea (mg/dL)	Serum creatinine (mg/dL)	WBC count (per mm^3^)	24 h serum Na^+^(mEq/L)	Fall in serum Na^+^(mEq/L)
1.	3.5	Bacteremia	0.18	132	13	0.2	21,200	130	2
2.	1.5	Hepatosplenomegaly with lymphadenopathy	0.45	135	18	0.2	11,600	131	4
3.	0.5	Bronchopneumonia	0.18	137	24	0.6	28,500	133	4
4.	0.33	Cerebral Palsy with breakthrough seizures	0.18	138	18	0.3	8,000	134	4
5.	2.75	West syndrome with seizures	0.18	138	18	0.2	28,200	134	4
**6.**	**5**	**Enteric fever with encephalopathy**	**0.18**	**142**	**15**	**0.2**	**24,100**	**133**	**9**
**7.**	**1.5**	**Simple febrile seizure**	**0.18**	**136**	**19**	**0.4**	**12,300**	**126**	**10**

*Bold font: patients with a potentially dangerous fall in serum sodium*.

The median fall in SNa was greatest for patients with a baseline SNa >140 mEq/L (Table 1), with the median fall for patients with a baseline SNa >145 mEq/L being 13 mEq/L.

Forty-two percent of children (24/56) experienced a rise in SNa at 24 h, and patients with mild baseline hyponatremia (SNa <135 mEq//L) experienced a median rise in SNa of 12 mEq/L, yet none developed hospital-acquired hypernatremia (SNa >145 mEq/L) (Table 1).

## Discussion

All the children in this cohort admitted to a general pediatric ward received hypotonic maintenance fluids, with the primary fluid prescribed being 0.18% NaCl (87.5%). While the mean serum remained unchanged during the study period, a significant number of patients (50%) experienced a fall in SNa and developed hospital-acquired or -aggravated hyponatremia (12.5%). This is significant because this study was specifically restricted to a group at relatively low risk for developing hyponatremia, by excluding patients who were critically ill, who were post-operative, who required bolus fluid therapy or who had underlying chronic diseases known to impair sodium and water homeostasis. The underlying diagnosis that placed patients at risk for elevated AVP levels and hyponatremia were primarily respiratory and central nervous system disorders.

We specifically evaluated the fall in SNa in patients whose presenting SNa was at or below the normal median sodium of 140 mEq/L, as their sodium would not be expected to fall in the absence of an impairment in free water excretion (Bold font in Table 1). Forty percent of patients (17/42) with a SNa ≤140 mEq/L experienced a fall in SNa. Patients with the highest SNa at presentation experienced the largest fall in SNa. One patient with SNa >145 mEq/L developed a-14 mEq/L fall in SNa, without developing hyponatremia. This large fall in SNa could be excessive in a patient at risk for increased intracranial pressure, such as meningitis. This data suggest that even low-risk children admitted to a general pediatric ward are at risk for hyponatremia. This also suggests that 0.18% NS is not an appropriate mIVF even in low-risk children as it can result in hyponatremia or an excessive fall in SNa.

While no child in this study developed hyponatremic encephalopathy, two children had a potentially dangerous fall in SNa of 9 and 10 mEq/L, respectively, while receiving 0.18% saline (Bold font in Table 2). This highlights the potential danger of administering hypotonic mIVF.

The mean SNa at baseline and 24 h remained unchanged, as the proportion of patients experiencing a rise and fall in SNa was similar. Patients with a baseline SNa <140 mEq/L generally experienced a rise in SNa and those with a baseline SNa >140 mEq/L experienced a fall in SNa, which would be the expected response to intravenous fluids provided that there is not a disorder in renal concentration or dilution. A fall in SNa when the baseline SNa is <140 mEq/L is not an expected response to fluid therapy and signifies and impairment in free water excretion.

At the time this study was conducted, there was a significant literature pointing to the dangers of hospital-acquired hyponatremia from hypotonic mIVF in high-risk children, i.e., post-operative, critically ill, and volume-depleted patients (1, 9, 14–16), but the risk in the general pediatric ward was not fully appreciated and the necessity of using isotonic fluids was debated (17). Since then, randomized prospective studies, including children admitted to a general pediatric ward, have demonstrated that they too are at risk for hospital-acquired hyponatremia from hypotonic mIVF and that isotonic mIVF are safe and effective in preventing hyponatremia in this group of patients (18–21). Recent NICE guidelines for fluid therapy in children now recommend isotonic mIVF (22). Despite this, there are those who still recommend hypotonic fluids (23).

Limitations of this study are that this is a single-center study, lacking a control group, and of a relatively small sample size. The finding in this study may not reflect that of other pediatric centers in India, and we are unable to assess how a similar group of patient would have fared if they received isotonic saline.

Epidemiological data regarding fluid-prescribing practices throughout the world are lacking, but surveys have suggested that hypotonic mIVF are the primary fluid used (24, 25). This study demonstrates that the current practice in a general pediatric ward in India, one of the most populous nations in the world, is to prescribe hypotonic mIVF. It also demonstrates that seemingly low-risk populations are at significant risk for developing hyponatremia. Our study lends support to the recent NICE fluid guidelines in children to avoid hypotonic mIVF, and highlights the need for general pediatricians to re-evaluate and possibly change mIVF practices.

## Author Contributions

SS and SB conceived and designed the study. SS conducted the study. SS and MM performed the data analysis, wrote and edited the manuscript.

## Conflict of Interest Statement

The authors declare that the research was conducted in the absence of any commercial or financial relationships that could be construed as a potential conflict of interest.
